# Use of routine hospital morbidity data together with weight and height of patients to predict in-hospital complications following total joint replacement

**DOI:** 10.1186/1472-6963-12-380

**Published:** 2012-11-01

**Authors:** George Mnatzaganian, Philip Ryan, Paul E Norman, David C Davidson, Janet E Hiller

**Affiliations:** 1 Faculty of Health Sciences, Australian Catholic University, Fitzroy, Victoria, Australia; 2 School of Population Health and Clinical Practice, Discipline of Public Health, The University of Adelaide, South Australia, Australia; 3 Data Management and Analysis Centre, The University of Adelaide, South Australia, Australia; 4 School of Surgery, University of Western Australia, Perth, Australia; 5 Emeritus Consultant Orthopaedic Surgeon, Royal Adelaide Hospital, South Australia, Australia

## Abstract

**Background:**

Routinely collected data such as hospital morbidity data (HMD) are increasingly used in studying clinical outcomes among patients undergoing total joint replacement (TJR). These data are readily available and cover large populations. However, since these data were not originally collected for the purpose of health research, a rigorous assessment of their quality is required. We assessed the accuracy of the diagnosis of obesity in HMD and evaluated whether the augmentation of HMD with actual weight and height of patients could improve their ability to predict major in-hospital complications following total joint replacement in men.

**Methods:**

The electronic records of 857 participants in the Health In Men Study (HIMS) who had had TJR were linked with Western Australia HMD. HMD-recorded diagnosis of obesity was validated using the actual weight and height obtained from HIMS. In-hospital major complications were modelled using multivariable logistic regressions that either included the actual weight and height or HMD-recorded obesity. Model discrimination was calculated using area under ROC curve.

**Results:**

The HMD failed to detect 70% of the obese patients. Only 64 patients (7.5%) were recorded in HMD as obese although 216 (25%) were obese [BMI: ≥30kg/m^2^] (sensitivity: 0.2, positive predictive value: 0.7). Overall, 174 patients (20%) developed an in-hospital major complication which was significantly higher in the overweight and obese comparing with patients with normal weight. HMD-recorded obesity was not independently associated with major complications, whereas a dose–response relationship between weight and these complications was observed (P=0.004). Using the actual weight and height of the participants instead of HMD-recorded diagnosis of obesity improved model discrimination by 9%, with areas under ROC curve of: 0.69, 95% CI: 0.64-0.73 for the model with HMD-recorded obesity compared with 0.75, 95% CI: 0.70-0.79 for the model with actual weight and height, P<0.001.

**Conclusion:**

Body weight is an important risk factor for in-hospital complications in patients undergoing TJR. HMD systems do not include weight and height as variables whose recording is mandatory. Augmenting HMD with patients’ weight and height may improve prediction of major complications following TJR. Our study suggests making these variables mandatory in any hospital morbidity data system.

## Background

Hospital morbidity data (HMD), or administrative claims data, are increasingly being used to study important clinical outcomes including in-hospital mortality [1,2], re-admissions [2,3], and post-operative complications [4]. These routinely collected data are both readily available and cover large populations offering advantages in regulatory and surveillance settings in that the data have been collected in a reasonably consistent manner over a number of years, and will continue to be collected, using similar procedures, into the future. However, in comparison with clinical data (usually retrieved from individual patient chart review) these data may lack detail on co-morbidities, severity scores, and timing of diagnoses [5-7]. Moreover, databases that have restricted coding spaces are often limited to a minimum set of data [8]. In addition, HMD do not routinely include important risk factors such as weight, height and detailed smoking history. Nonetheless, owing to their many advantages, researchers have tried to improve these data, validate them [9,10], and augment them with additional information in order to use them in health care research [11].

Total joint replacement (TJR) is among the most common elective surgical procedures performed in developed countries [12]. The incidence of this procedure has risen over recent years mainly because of the ageing population and increases in the prevalence of risk factors such as obesity [13]. It has been estimated that the demand for total joint replacement will continue to grow [12]. Although primary total joint replacement is considered one of the safest and most effective surgical procedures [14], the procedure is nevertheless associated with short- and long-term complications that can also be life-threatening [15,16]. These adverse outcomes are more frequent in older patients [16], particularly men [15], and in the obese [3,17,18], and a thorough understanding of potential complications in these groups is important for the delivery of high quality and safe medical care. To study these outcomes, researchers have used existing large databases including joint replacement registries and hospital morbidity data. The latter have frequently been used to characterize the rates of immediate postoperative outcomes of both primary [15-18] and revision total joint replacement [15,19]. Methods to improve existing data sources, such as HMD, to predict complications following TJR have never been documented.

In an earlier analysis, we have shown that major comorbidities (such as myocardial infarction and cancer) and major operations (such as TJR and coronary artery bypass graft surgery) are more likely to be recorded in the Western Australia (WA) HMD than conditions of less serious nature such as dyslipedemia [9]. In this current study, we assessed the validity and recording of the diagnosis of obesity in this HMD system, and we evaluated whether its augmentation with actual weight and height (both measured by clinical staff) could improve its ability to predict major in-hospital complications following TJR.

## Methods

### Data sources and study population

The study integrated longitudinal data from a large population-based cohort with WA HMD. The study population is drawn from the Health In Men Study (HIMS) which arose from a randomized trial of ultrasound screening for abdominal aortic aneurysm in men aged 65–83 living in Perth, Western Australia [20]. Of the 19,352 men who were invited, 12,203 (63%) attended the baseline screening in 1996–9. At baseline, the participants provided detailed health and other information including a comprehensive smoking history. In addition, study nurses recorded the individuals’ weight and height. During 2001–04, the surviving men of the 12,203 initial participants were invited to a follow-up study during which they were weighed a second time. All men were followed from baseline screening until they experienced their first TJR or died or were right censored at the end of follow-up (March, 2007) [21]. Electronic record linkage was used to identify admissions to hospital (hospital morbidity data) for TJR (Table 1) and post-operative complications in the target population. Of the total 12,203 men, 857 (7%) had a total joint replacement after baseline screening and these constituted the study population for this analysis.

**Table 1:** ICD-9 and ICD-10 codes used to detect primary total hip or total knee replacement

**ICD version**	**Code**	**Description of procedure**
ICD-9-CM	81.51	Total hip replacement
	81.54	Total knee replacement
ICD-10-AM*	49318-00	Total arthroplasty of hip, unilateral
	49319-00	Total arthroplasty of hip, bilateral
	49518-00	Total arthroplasty of knee, unilateral
	49519-00	Total arthroplasty of knee, bilateral
	49521-00	Total arthroplasty of knee with bone graft to femur, unilateral
	49521-01	Total arthroplasty of knee with bone graft to femur, bilateral
	49521-02	Total arthroplasty of knee with bone graft to tibia, unilateral
	49521-03	Total arthroplasty of knee with bone graft to tibia, bilateral
	49524-00	Total arthroplasty of knee with bone graft to femur and tibia, unilateral
	49524-01	Total arthroplasty of knee with bone graft to femur and tibia, bilateral
	49534-01	Total replacement arthroplasty of patellofemoral joint of knee

*The ICD-10 codes were based on those listed in the database.

The HMD system is a core part of the WA Linked Data System [22] and includes demographic, diagnostic, and procedural information on all patients discharged from all public and private hospitals in WA. The HMD allow the inclusion of up to 21 diagnoses and 11 procedure codes for each hospitalization. In an earlier validation study, we have shown that the sensitivity and positive predictive value of the HMD-recorded TJR were both 0.92 and the specificity was 0.98 [9].

### Statistical analysis

#### Validity analysis

The diagnosis of obesity was retrieved from the HMD using the following codes: the International Classification of Disease, 9th Revision, Clinical Modification (ICD-9-CM) “278.0” code and the ICD-10-AM (Australian Modification) “E66” code. Validation of this HMD-recorded diagnosis of obesity was performed using the body mass index (BMI) that was calculated from the actual weight and height of the participants (obtained from HIMS baseline survey). Those who had a BMI of 30 kg/m^2^ or more were considered to be obese and this was held as the “Criterion Standard”. The sensitivity and positive predictive value (PPV) were based on a 2x2 table (having a recorded diagnosis of obesity in HMD yes/no versus BMI ≥ 30kg/m^2^ yes/no).

#### Weight measured at baseline and follow-up

Available data did not permit us to account for weight change over time. Hence we used body weight of the participants that was measured at baseline. Time to TJR was not long (mean 4.6 (SD 2.7) years) and, therefore, we assumed that weight measured at baseline (1996–9) remained constant up till surgery. To test this assumption, we compared the weights measured at baseline with the corresponding weights measured 5 years later in a HIMS follow-up survey conducted in 2001–4. Of all men who had had TJR, 56% participated in both baseline and follow-up HIMS surveys. The mean change in weight in kilograms was −0.13 (SD 4.1) [range −15.9 to 22.4]. No significant differences in weight change were observed over the period of 5 years. Agreement between the weights was also demonstrated in the Bland-Altman plot (as reported previously [18]) which supports our assumption of relatively constant weight over time in this cohort of older men.

#### Classification of complications

All 857 men who had a TJR were followed till hospital discharge. All conditions recorded in HMD were retrieved from the index-TJR admission. If a certain condition was recorded in previous hospital admissions (other than the index admission), it was regarded as a co-morbidity rather than a complication - a method that increased the specificity of the diagnosis rather than its sensitivity. The detected complications were further clinically classified as major or minor based on a survey of 13 experienced orthopaedic surgeons. The surgeons were approached by mail and were asked to classify each of the 60 reported conditions into major or minor and all 13 participated provided complete responses [18]. The surgeons were blinded to the outcome of these diagnoses. The only information that was provided was the overall mean age and gender of the study population. A complication that was potentially life-threatening was defined as major, while a complication that did not threaten life but did demand medical intervention was defined as minor [23]. Inter-rater agreement was calculated using kappa coefficient and the final decision to classify a condition into major or minor followed a majority rule.

#### Risk of major complications

In a first model (Model 1), risk of an in-hospital major complication was assessed using a multivariable logistic regression that was fitted to the data as a function of age, Charlson Co-morbidity Index (CCI) [24], fracture of lower limb, obesity diagnosis as recorded in HMD (a dichotomous variable of yes or no), years of smoking, socioeconomic status based on Socio-Economic Index For Areas (SEIFA) [25], number of past hospitalizations, insurance payer type (public versus private hospitals), type of TJR (total hip replacement [THR] or total knee replacement [TKR]) and presence of a minor complication. SEIFA indices indicate relative social disadvantage of populations living in different geographic areas with low scores reflecting disadvantage. A second model (Model 2) was fitted to the same variables as the first model, except for HMD-recorded obesity that was substituted with actual weight and height of study participants (obtained from baseline HIMS study). In Model 2, weight was introduced either as a continuous variable, or as quintiles categorized according to the weight distribution in the cohort while height was introduced as a continuous variable. The categorization of weight into quintiles was done in order to investigate the presence of any dose–response effect with any increase in the weight category. Model discrimination for each of the models was calculated using area under ROC curve. The original Charlson weights [26] were applied to calculate the Charlson Co-morbidity Index.

Ethical approval was obtained from the Human Research Ethics Committees of Health Department of Western Australia (October 12, 2009; AHEC EC00422) and The University of Adelaide (August 10, 2009; H-106-2009) prior to commencement of the study. All analyses that used de-identified data were performed using Stata statistical program (version 11, Stata-Corp.).

## Results

### Validity of HMD-recorded obesity

Of the 857 men (mean age at surgery 76.3 [SD 4.6] years) who had had a TJR, 488 (57%) were overweight [BMI: 25–29.9 kg/m^2^] and 216 (25%) were obese [BMI ≥30 kg/m^2^]. Of the latter 216 patients, only 64 men (30%) were recorded as obese in the HMD which failed to detect 70% of the obese patients. The sensitivity of HMD-recorded diagnosis of obesity was 0.2 and its corresponding positive predictive value was 0.7. Compared with patients with normal weight [BMI: 18.5-24.9kg/m^2^] (based on actual weight and height from HIMS survey), the obese were significantly younger (P<0.001) and belonged to a lower social economic status (P=0.03) (Table 2). However, these differences in patients’ characteristics were not apparent when the patients were stratified according to HMD-recorded diagnosis of obesity. The main differences in the characteristics of those with and without a HMD-recorded diagnosis of obesity were the significantly higher Charlson Co-morbidity Indices and higher duration of smoking among those with a recorded diagnosis of obesity.

**Table 2:** Characteristics of patients by obesity diagnosis as recorded in hospital morbidity data and by body mass index based on actual weight and height measured by nurse

**Patient characteristic**	**Diagnosis of obesity as recorded in HMD**^**1**^		**Body mass index calculated from weight and height measured by nurses from HIMS survey**^**2**^		
	**No obesity diagnosis N=793 (92.5%)**	**With obesity diagnosis N=64 (7.5%)**	**BMI 18.5-24.9 N=153 (18%)**	**BMI 25-29.9 N=488 (57%)**	**BMI>30 N=216 (25%)**
Age, mean (SD)	76.3 (4.6)	75.3 (4.3)	77.1 (4.8)	76.4 (4.6)	75.4 (4.4)!!
CCI, mean (SD)	1.2 (1.7)	2.3 (2.0)!	1.4 (2.0)	1.2 (1.6)	1.4 (1.7)
SES, %					
Low	30%	27%	26%	28%	36%
Middle	32%	39%	30%	32%	33%
High	38%	34%	44%	39%	31%!
Yrs of smoking, mean (SD)	21.3 (19.8)	28.4 (19.2)!	19.3 (20.2)	21.9 (19.6)	23.4 (19.8)

! 0.001<p<0.05; !! p<0.001.

^1^ Patients with an obesity diagnosis in HMD were compared with those who had no such diagnosis in HMD.

^2^ Patients with BMI 25-29.9 or BMI > 30 were compared with those with BMI 18.5-24.9.

Abbreviations: CCI (Charlson Co-morbidity Index); SES (socioeconomic status according to distribution of Socio Economic indices For Areas (SEFA); Yrs (years)).

### In-hospital complications

The overall inter-rater agreement between the surgeons who classified the complications into major or minor was moderate [18]. A total of 174 patients (20%) developed an in-hospital complication that was classified as major (Table 3). An increased risk of these complications was detected both in patients with a HMD-recorded diagnosis of obesity and in patients whose actual BMI was 25 or more (Table 4). However, when stratified by Charlson Co-morbidity Index categories, the differences in the rates between those with and without a HMD-recorded obesity became statistically insignificant. This was not apparent when the stratification was done by the actual BMI categories.

**Table 3:** **List of in-hospital complications**^**1 **^**following an elective TJR (as reported in HMD during index admission) classified as major by 13 orthopedic surgeons by body system (N=857)**

	**Number**	**Percentage**
**Cardiovascular**		
Acute myocardial infarction	6	0.7
Arterial embolism	1	0.1
Cardio respiratory arrest	4	0.5
Angina pectoris / unstable angina	9	1.1
Complete heart block	1	0.1
Congestive heart failure	10	1.2
Post operative shock	1	0.1
Supra-ventricular / ventricular tachycardia	6	0.7
Thromboembolism / deep vein thrombosis	17	2.0
**Respiratory**		
Acute pulmonary edema	3	0.4
Adult respiratory distress syndrome	5	0.6
Pneumonia / aspiration pneumonia	8	0.9
Pulmonary embolism	12	1.4
**Gastrointestinal**		
Abdominal obstruction	14	1.6
Acute gastrointestinal bleeding / ulcer	9	1.1
Acute hepatic failure	1	0.1
**Renal**		
Acute renal failure	15	1.8
Oliguria / anuria	16	1.9
**Musculoskeletal**		
Dehiscence of surgical wound	2	0.2
Hemorrhage complicating a procedure	22	2.6
Hip abscess / septic arthritis / acute osteomyelitis	3	0.4
Mechanical complications due to prosthesis (e.g., fracture of bone)	9	1.1
**Neurological**		
Acute cerebrovascular accident / transient ischemic attach	4	0.5
Convulsions	1	0.1
Semi coma	1	0.1
**General**		
Bacteremia	15	1.8
Diabetic hypoglycemic shock	2	0.2
Post operative infection / sepsis	21	2.5

^1^A person may have more than one complication.

**Table 4:** Rates of major in-hospital complications by HMD-recorded obesity and body mass index based on actual weight and height of patients by Charlson Co-morbidity Index categories

**Charlson Co-morbidity Index categories**		**Diagnosis of obesity as recorded in HMD**^**1**^		**Body mass index calculated from weight and height measured by nurses from HIMS survey**^**2**^		
		**No obesity diagnosis N=793**	**With obesity diagnosis N=64**	**BMI 18.5-24.9 N=153**	**BMI 25-29.9 N=488**	**BMI>30 N=216**
0	n=384	14%	33%	17%	13%	17%
1-2	n=323	23%	32%	10%	28%!	25%!
> 3	n=150	25%	33%	6%	33%!	31%!
**All**	**n=857**	**19%**	**34%!**	**12%**	**22%!**	**23%!**

! 0.001<p<0.05.

^1^ Patients with an obesity diagnosis in HMD were compared with those who had no such diagnosis in HMD.

^2^ Patients with BMI 25-29.9 or BMI > 30 were compared with those with BMI 18.5-24.9.

**Figure 1:**
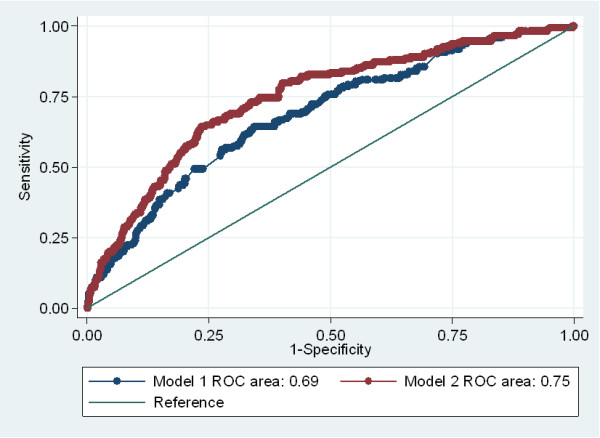
Areas under receiver operating characteristic (ROC) curves of multivariable logistic models that included HMD-recorded diagnosis of obesity (Model 1) or actual weight and height of patients (Model 2).

Adjusting for age, Charlson Co-morbidity Index, socio-economic status, duration of smoking, type of joint replacement, fracture of lower limb, number of past hospital admissions, type of hospital and presence of a minor complication, no statistically significant associations were found between HMD-recorded obesity with risk of major complications following TJR as shown in Model 1 in Table 5, whereas, a dose–response effect between actual weight and risk of major complications was observed (Model 2 in Table 5). A test for trend in the log odds-ratios across weight quintiles yielded P=0.004. Using the actual weight and height of the participants instead of HMD-recorded diagnosis of obesity improved model discrimination by 8.7%, with areas under ROC curve of: 0.69, 95% CI 0.64-0.73 in Model 1 compared with 0.75, 95% CI 0.70-0.79 in Model 2, P<0.001 (Figure 1). Using weight as a continuous variable instead of weight quintiles in Model 2 produced similar findings in model discrimination (results not shown).

**Table 5:** Risk of major in-hospital complication following primary TJR: multivariable logistic regressions using either HMD-recorded obesity (model 1) or actual body weight and height (model 2)

	**Multivariable analysis**		**Multivariable analysis**	
	**Model 1**		**Model 2**	
	**OR (95% CI)**	***P *value**	**OR (95% CI)**	***P *value**
**Obesity as recorded in HMD**	1.6 (0.9 - 2.9)	0.1	**-**	**-**
**Weight quintiles, kg**	-	-		
1st quintile: < 73.2 (ref)			1.0	
2nd quintile: 73.3-79.6			1.2 (0.7 - 2.3)	0.5
3rd quintile: 79.7-84.4			1.7 (0.9 - 3.1)	0.1
4th quintile: 84.5-91.8			1.9 (1.0 - 3.4)	0.04
5th quintile: > 91.9			2.3 (1.2 - 4.4)	0.01
Area under ROC curve:	0.69		0.75	

The models also controlled for age, Charlson Co-morbidity Index, socioeconomic status, height, type of replacement, fracture, years of smoking, presence of a minor complication, number of past hospitalizations, and private or public hospital.

## Discussion

In a cohort of men who had had a primary TJR, we found that actual weight independently predicted major in-hospital complications following the procedure showing a dose–response effect, whereas a record of obesity diagnosis in hospital morbidity data did not. Adding actual weight and height to a HMD system makes the latter a better prognostic tool for this major health outcome.

The utility of hospital morbidity data as a resource for medical research has been keenly investigated in recent years [1-11]. While clinical data usually retrieved from patients’ files are considered the gold standard for accurate clinical information, these are costly and time consuming to obtain and often large clinical databases for comparative purposes are not easily available. Therefore, claims data or HMD are being increasingly used to assess clinical outcomes, and monitor, evaluate, and improve the quality of care. However, differences in HMD-based-outcome among patients may or may not indicate differences in quality of care that the patients received because these differences may be attributed to many factors including differences in age and co-morbid conditions, but also differences in the quality of the data [5-7]. Since the ability of these routinely collected data to predict adverse outcomes may largely depend on the extent and accuracy of the data on each patient’s clinical condition when care began, researchers have tried to validate, improve and augment them with additional information in order to use them in health care research. Increasingly, studies show how the augmentation of administrative data with minimal clinical information may improve the former’s predictive power [27,28]. In a retrospective study of 46,769 patients in 30 acute care hospitals, Pine et al. demonstrated how the addition of laboratory data to hospital administrative datasets could provide accurate predictions of inpatient mortality from acute myocardial infarction, cerebrovascular accident, congestive heart failure or pneumonia with significant improvements in models’ discrimination [27]. Another study [28] showed how models using claims data to predict mortality following cardiac bypass surgery can be improved with the addition of minimal clinical variables. Methods to improve HMD to predict complications following TJR have never been documented and this was the focus of our current study.

Postoperative complications following a total joint replacement procedure are not uncommon in elderly patients and in the obese [3,15-18]. The impact of obesity on surgical outcomes is achieving significant attention because of the rapidly increasing prevalence of this condition worldwide [29]. In our elderly cohort, 25% of the patients who underwent TJR were obese. However, the WA HMD failed to report this condition among 70% of our obese study population. In earlier analyses, we have shown that body weight is an important risk factor for various adverse outcomes in patients undergoing TJR. We found that, compared with patients with normal weight, the overweight or obese were significantly more likely to develop in-hospital major complications [18], to stay longer in hospital, and to be readmitted within 5 years of this procedure [3]. Nevertheless, HMD systems do not include the weight and height of patients as variables whose recording is mandatory. In this analysis, we found that obesity was under-reported in HMD and was selectively recorded for more severely ill patients. When assessing postoperative complications, HMD alone produced inferior predictive models compared with those that also accounted for the actual weight and height of the patients. The inclusion of actual weight and height in the HMD makes the HMD a better prognostic tool to assess major complications among patients undergoing TJR.

Strengths of this study include its population-based provenance, the longitudinal design and the integration of clinical data with validated HMD. For each participant, any significant morbidity or health-related outcome was retrieved from the linked data in the period 1970 through to 2007 and this enabled us to better account for patient co-morbidities. However, the study has some limitations. HMD may not differentiate complications from co-existing conditions [30]. Our method of retrieving (from the TJR-index admission) only the diagnoses that were reported for the first time for every patient may have misclassified some diagnoses as co-morbidities. Furthermore, HMD systems may be disadvantaged by under-coding or over-coding. We had no access to patients’ charts and, therefore, we could not validate these conditions against these charts. Moreover, classification of a complication as major or minor may differ among studies and our data did not allow us to assess risk of individual conditions. This study also did not account for other surgical and intervention-related factors (such as type of anesthesia) that may also be associated with postoperative complications.

## Conclusions

Body weight is an important risk factor for numerous health outcomes and there is increasing evidence to support a correlation between obesity and adverse outcomes in patients undergoing a TJR. The lack of validity of the HMD-recorded diagnosis of obesity limits its use in health research. This study is the first to report that adding actual weight and height to HMD may significantly improve the model discrimination for major complications in an elderly patient population. Since the standard hospital practice is to measure the weight and height of patients [31], our study suggests making actual weight and height mandatory variables in any hospital morbidity data system. Identification of patients who are at increased risk for developing postoperative complications following TJR may assist hospitals in assessing casemix, quality of care, and resource allocation, as well as this may assist clinicians in selecting patients for surgery, and informing patients about their individual level of risk.

## Competing interests

The authors declare that they have no competing interests.

## Authors’ contributions

All authors were involved in drafting the article or revising it critically for important intellectual content, and all authors approved the final version to be published. Study conception and design: (Mnatzaganian, Ryan, Hiller.); Acquisition of data: (Norman.); Analysis and interpretation of data: (Mnatzaganian, Ryan, Davidson, Hiller.). All authors read and approved the final manuscript.

## Pre-publication history

The pre-publication history for this paper can be accessed here:

http://www.biomedcentral.com/1472-6963/12/380/prepub
